# Parental Participation in the Environment: Scale Validation Across Parental Role, Income, and Region

**DOI:** 10.3389/fpsyg.2022.788306

**Published:** 2022-03-04

**Authors:** Fanli Jia, Angela Sorgente, Hui Yu

**Affiliations:** ^1^Department of Psychology, Seton Hall University, South Orange, NJ, United States; ^2^Department of Psychology, Università Cattolica del Sacro Cuore, Milan, Italy; ^3^School of Education, Harbin Normal University, Harbin, China

**Keywords:** environmental behavior, validation, parent socialization, parent participation, scale, intergenerational transmission

## Abstract

Parental participation has gained significant attention in environmental psychology, which has revealed a need for an instrument that can measure parental participation with children regarding environmental issues. The present study met this need by validating the parental participation in the environment (PPE) scale. This process began with 45 Chinese parents participating in an individual interview and group discussions, which helped generate a list of eighteen parent-child environmental activities. The activities were then modified and validated in the current study with a diverse group of 969 parents recruited from six major Chinese cities. Both score structure evidence and generalizability evidence were obtained within this sample, and psychometric tests suggested a single factor construct with nine items. Once the PPE scale was revised, it showed measurement invariance across the parent who responded to the items (mother vs. father), across the child’s primary caregiver (mother vs. father vs. grandparent), across the family’s living region (North China vs. South China), as well as across the family’s income group. Finally, evidence based on relations to other variables showed a relationship among parents’ PPE, pro-environmental behavior, and connectedness with nature. As a result, the study provided a novel measure to assess pro-environmental socialization *via* parental participation.

## Introduction

A parent is an essential social agent who can influence children’s beliefs, values, and behaviors (Brim, 1966; Bandura, 1977; Zigler and Seitz, 1978; Bronfenbrenner, 1979). Recently, researchers have demonstrated the connection between parental socialization and parents’ and children’s pro-environmental behaviors (e.g., Grønhøj and Thøgersen, 2017; Katz-Gerro et al., 2020; Gong et al., 2021; Jia and Yu, 2021). Overall, research has agreed that daily parental pro-environmental behaviors influence children’s understanding of human-nature relationships and predict children’s pro-environmental intentions. However, most researchers have only assessed parental behaviors as a proxy of their direct involvement and have presumed that parents’ behaviors could be conveyed to their children. How parents interact with their children toward environmental issues has not been fully explored. This gap is partially due to the lack of an instrument to measure parental participation in environmental issues. As a result, this study validated a parental participation in the environment (PPE) scale using a group of parents recruited from different demographic variables.

Research in environmental psychology has documented the significant role that parental pro-environmental behavior has in their children’s behavioral outcomes. Grønhøj and Thøgersen (2009) found a positive relationship between parents’ and children’s pro-environmental behaviors, including purchasing green products, conserving energy, and sorting waste. Grønhøj and Thøgersen (2012) further demonstrated that parents could transmit their values, beliefs, and behaviors to their children. However, other research has revealed that the direct intergenerational transmission of ecological behavior is not always the case. For example, Matthies et al. (2012) found that parents’ pro-environmental behavior of reusing paper did not convey to their children. Similarly, Jia and Yu (2021) found no correlation between parents’ and children’s energy conservation behavior (e.g., turning off the heater, air conditioner, and air purifier). These authors argued that there may be an unstable intergenerational transmission of pro-environmental behavior from parents to their children. The inconsistency may be explained by the lack of family norms and direct engagement regarding environmental issues. For example, some parents may not explain the environmental benefits of reusing paper or turning off large appliances (e.g., heater, air conditioner, humidifier) to their children. Conversely, children might not be able to observe their parents’ actions directly to learn from them. Therefore, parents’ and children’s PEBs might have an unstable relationship due to their direct interaction (family norms, discussion, or engagement). Recent studies have focused on family socialization in environmental issues to solve the problem of unstable intergenerational transmission.

Several recent investigations have started to further explain how a parent’s environmental worldview and behavior can be conveyed to their child *via* parental socialization (e.g., parental norms, parenting styles, parent-child relationships, and parental control). For example, Grønhøj and Thøgersen (2017) found that adolescents’ perceptions of autonomy support from parents were positively associated with their environmental behaviors. Collado et al. (2019) found that parental norms predicted adolescents’ environmental behaviors. Gong et al. (2021) found that parents’ green consumption values predicted children’s environmental values only if there was a positive parent-child relationship. Ando et al. (2015) found that parental seriousness, norms, and perceived behavior control mediated parents’ and children’s environmental behavior.

Although previous studies have shown that different aspects of parental socialization influence children’s environmental worldviews, very few studies have directly examined PPE. According to the parental socialization theory (Grusec, 2011; Grusec and Davidov, 2019), one important domain of parental socialization is group participation (e.g., parental participation). Through this socialization, parents interact with children, model learning, and organize activities so that parents can provide meaningful opportunities for their children (Grusec, 2011; Grusec and Davidov, 2019). Rogoff et al. (2007) argued that by intentionally observing parental behaviors, children would anticipate exhibiting those behaviors themselves in the future. Thus, parental participation (e.g., parent-child interaction through family activities) helps children internalize societal values as well as learn social customs and behaviors (Rogoff et al., 2007).

Two recent studies explored the role of parental participation in the intergenerational transmission of pro-environmental behaviors. Katz-Gerro et al. (2020) compared the intergenerational transmission of environmental behaviors with various domains of parental socialization (e.g., reciprocity, guided learning, sense of control, and parental participation). They found that parental participation was the only domain that predicted children’s environmental behaviors, including living a sustainable lifestyle, saving energy, and reducing waste. In addition, Jia and Yu (2021) investigated parental socialization *via* family norms, parent-child discussions, and parent-child engagement, and they found that parent-child engagement was the strongest mediator between parents’ and children’s pro-environmental behaviors. They suggested that parents must be directly involved in their children’s various pro-environmental behaviors and environmental worldviews so that children can directly observe the behavior and its value (Jia and Yu, 2021). Therefore, parents’ participation with their children in environmental issues appears to be crucial for ensuring intergenerational transmission of pro-environmental behaviors.

However, the previous studies on parental participation did not use a validated measure. For example, Katz-Gerro et al. (2020) adapted four items from a theoretical paper on five domains of socialization to measure parent-child group participation. Jia and Yu (2021) developed 18 items to measure parent-child environmental interaction without thorough validation in a representative sample. Therefore, it is important to examine parental participation with their children in the environment directly and to consider what environmental issues, activities, or human-nature interactions have not yet been studied. Since these gaps are partially due to the lack of an instrument to assess parental participation, the present study developed and validated an instrument that could assess them.

The current study modified and validated the items from Jia and Yu’s (2021) study using a large sample of parents in China. This scale provides a general worldview of environmentalism by examining parents’ involvement in pro-environmental activities, including waste management. It is worth noting that Municipal Solid Waste Management Regulation (e.g., recycling for bottles, cardboard, kitchen waste, electronic waste) was just implemented in China in 2019 (Zhou et al., 2019; Xiao et al., 2020). These city waste management regulations require each household to sort “recyclable waste,” “hazardous waste,” “wet waste,” and “dry waste” into four waste collection bins at a particular time and location (Shanghai Municipal People’s Government [SMPG], 2018; Ministry of Housing and Urban-Rural Development of the People’s Republic of China, 2019). Community and city volunteers “supervise” the waste-sorting sites, and they have the authority to fine residents for not sorting the waste appropriately (Lu and Sidortsov, 2019; Zhou et al., 2019). This new regulation provides a great opportunity for parents to involve their children in their daily activities (e.g., sorting waste together).

In the field of environmental psychology, many researchers have indicated that demographic variables (e.g., geographical area and socioeconomic status) should be examined rather than “statistically controlled” (e.g., Gifford and Nilsson, 2014; Medina et al., 2019). Two recent studies investigating pro-environmental behaviors in North and South China have provided preliminary evidence of why this is important. Firstly, Yu (2014) examined one city from Northern China (Heilongjiang) and one city from Southern China (Ningxia) and found North-South differences regarding residents’ environmental attitudes. The difference in attitudes between these two geographical regions was demonstrated by examining residents’ lifestyles and resources in each region. Secondly, Jia and Yu (2021) pointed out that South China residents purchase portable heaters that they can adjust manually in their homes. In contrast, North China residents cannot adjust the heat in their homes because they have a centralized heating service instead. Since parents in North China do not control their heating system, they are unlikely to teach their children to conserve heating energy. Thus, parents from North and South China may become involved in different kinds of environmental activities with their children.

In addition, socioeconomic status (SES) should be considered when examining individuals’ environmental behaviors (e.g., Gifford and Nilsson, 2014; Pampel, 2014; Schuldt and Pearson, 2016; Medina et al., 2019). For example, environmental activists tend to be of higher SES (Medina et al., 2019). Parents with a higher SES may be more likely to demonstrate environmental activism because they can access more educational opportunities to understand environmental values better and purchase more expensive, environmentally friendly products (Jones and Dunlap, 1992; Gifford and Nilsson, 2014). Furthermore, it is reasonable to assume that parents’ SESs affect what resources they can provide their children. For example, high-income families may have more green space in their neighborhoods than people living in low-income areas (Castonguay and Jutras, 2009; Tillmann et al., 2018). A recent study indicated that even free neighborhood parks are less likely to be used in low-income areas than in high-income areas (Cohen et al., 2016). Moreover, high-income parents may be more likely to engage with children in costly leisure activities (e.g., visiting national parks, local zoos, and field trips) to learn about nature than low-income parents who cannot afford them.

## Current Study: Scale Validation and Modification

In the current study, we developed and validated a scale that can assess PPE for use in China. To develop the scale, we conducted online interviews with parents from North and South China to compile a list of environmental activities that they had been involved in with their children. All the activities were narrowed down to a list of PPE items. Then, we conducted psychometric tests to verify if the newly developed instrument yielded valid scores.

We collected *score structure evidence* to test the scale’s factorial structure and verify that each factor was a reliable measure of the construct. Second, we collected *generalizability evidence* to verify if the scale measurement model was invariant across the parent who responded to the items (mother vs. father), across the child’s primary caregiver (mother vs. father vs. grandparent), across the family’s living region (North China vs. South China), as well as across the family’s income group (less than 5,000 CNY vs. 5,000–10,000 CNY vs. 10,000–20,000 CNY vs. more than 20,000 CNY).

We also collected validity evidence to test the relationship between the PPE scores and constructs that should be associated, according to the general literature (i.e., parent’s pro-environment behavior and connectedness with nature). Past studies indicated that parental socialization (parent-child interaction) should be positively associated with pro-environmental behavior (Katz-Gerro et al., 2020; Jia and Yu, 2021). Additionally, using an eco-psychology perspective on the importance of human-nature interaction, Nisbet et al. (2009) found that connection with nature had a direct effect on an individual’s pro-environmental behavior. Thus, parent-child involvement in environmental issues should also be positively associated with their affinity for nature, therefore engaging them in environmental protection.

In a previous study, an initial list of parent-child activities was developed using a two-step interview approach (Jia and Yu, 2021). First, parents were asked to list environmental activities that they had been involved in with their children. Then the parents were interviewed in a small group setting. This approach has also been used in other studies to develop an initial list of items (e.g., Krettenauer et al., 2016; Jia et al., 2019). A brief description of the procedure can be found in the previous article (Jia and Yu, 2021). The current article provides a detailed description of the procedure in Supplementary Appendix A.

We intended to validate the 18 items to verify if the newly developed instrument yielded reliable and valid results in a representative sample. Score structure evidence (e.g., item analysis, exploratory factor analysis, and confirmatory factor analysis) and generalizability evidence (i.e., measurement and structural invariance across Chinese parents in different regions, income groups, and caregiver roles). At the end, we tested the validity evidence based on relations to parental pro-environmental behavior and connectedness with nature.

## Materials and Methods

### Participants

Data were collected from 969 Chinese parents (75.6% mothers) whose children were in 1st to 6th grade. Respondents’ ages ranged from 25 to 66 years (*M* = 36.91; *SD* = 6.52). The participants were recruited from local summer schools in North and South China (Beijing/Heilongjiang in the North; Guangzhou, Fujian, Zhejiang, and Henan in the South). Twenty-eight summer schools were contacted based on the third author’s personal connection. All parents filled out an online survey during their free time. Consent forms were obtained, and a unique ID was assigned to each parent. The study was approved by the Ethics and Research Board at the third author’s institution in China.

### Measures

In the survey, participants filled in items about demographic variables referring to the respondent (age, parental role) and his/her family (Where do you live with your family? What is your family’s income? Who is your child’s primary caregiver?). They also completed the following measurement scales:

#### Parental Participation in the Environment

This scale was developed to assess the frequency of parental participation in environmental activities with their children. It is composed of 18 items that were developed in the preliminary parental interview study. Using a 5-point scale, parents were asked to indicate the frequency (*1 = Never to 5 = Always*) with which they do specific environmental activities with their children (e.g., “Separate the trash with your children”). The full list of items is reported in Supplementary Appendix B.

#### Pro-Environmental Behavior

The Chinese version (Krettenauer et al., 2020) of the pro−environmental behavior scale developed by Krettenauer (2017) was used to assess the levels of parents’ pro-environmental behaviors. Samples of the 14 items we adopted are “I turn off TV and computer screens when they are not in use” and “I prefer using environmentally friendly products at home.” Participants responded to these items using a 5-point Likert scale from *1 = Never to 5 = Always.* We confirmed the mono-dimensional structure of the scale [χ^2^(71) = 345.823; *p* < 0.001; RMSEA = 0.068 [0.061, 0.075]; CFI = 0.89; SRMR = 0.042] and its reliability (ω = 0.79). The full list of items is reported in Supplementary Appendix C.

#### Connectedness With Nature

The 6-item connectedness with nature scale was adapted from Collado et al. (2013) and was used in a study by Liu and Chen (2018) to measure the emotional affinity with nature in a Chinese sample. Respondents answered this scale using a 5-point Likert scale (*1 = Totally Disagree to 5 = Totally Agree*). Sample items are “I feel free and relaxed in nature” and “I feel that in nature, my life can be rich and colorful.” We confirmed the mono-dimensional structure of the scale [χ^2^(6) = 16.584; *p* < 0.001; RMSEA = 0.046 [0.020, 0.073]; CFI = 0.99; SRMR = 0.010] and its reliability (ω = 0.91). The full list of items is reported in Supplementary Appendix D.

### Data Analysis

#### Data Cleaning and Descriptive Analysis

Firstly, we checked data for missing data mechanisms and removed outliers. In particular, we ran Little’s MCAR test to verify if missing data were completely random (MCAR; Little, 1988). Univariate outliers were checked by examining the z scores of all the continuous variables and deleting subjects who had absolute values of the z scores exceeding 3.29 (Tabachnick and Fidell, 2013). Multivariate outliers (participants who exceeded the critical values based on the chi-square distribution of the Mahalanobis distance [*p* < 0.001]) were detected and removed using the Mahalanobis distance (Tabachnick and Fidell, 2013). All these analyses were run using SPSS software.

#### Score Structure

The sample was randomly divided into two sub-samples. The first one was used to identify items to retain in the final version of the scale. In particular, item analysis and Exploratory Factor Analysis (EFA) were performed to detect items with psychometric characteristics sufficiently good enough to be retained as items on the scale, following the procedure suggested by Sorgente and Lanz (2019). Descriptive statistics and EFA (performed using Maximum Likelihood as the extraction method, Kaiser as the criterion for factor selection, and Oblimin as rotation method) were performed with SPSS software to evaluate several characteristics of each item: (1) response rate (items with missing data above 30% were discarded; Crocker and Algina, 1986); (2) normal distribution of answers to that item (items with kurtosis and skewness higher than | 1| were removed from the scale; Muthén and Kaplan, 1985); (3) correlation of the item with other items (items with initial or extraction communality lower than 0.40 were removed from the scale; Fabrigar et al., 1999); (4) whether the item loaded on just one factor (items having all factor loadings lower than 0.30 in EFA and/or being multifactorial were removed; Fabrigar et al., 1999); and (5) the factor’s internal consistency (items that decrease the internal consistency of the factor they load on were removed from the scale).

After removing inadequate items according to the previous criteria, a Confirmatory Factor Analysis (CFA) was performed in Mplus with the same sub-sample to confirm the validity of the factorial structure identified by the EFA. The CFA also helped identify problematic items, according to the Mplus modification indices (i.e., indices which indicate how much the χ^2^-value would drop if a specific modification was applied to the model). In particular, the comparative fit index (CFI), root mean square error of approximation (RMSEA), and standardized root mean square residual (SRMR) were used to assess absolute model fit. CFIs equal to or higher than 0.90 and RMSEAs and SRMRs equal to or lower than 0.08 indicated an acceptable fit. CFIs equal to or higher than 0.95 and RMSEAs and SRMRs equal to or lower than 0.05 indicated a good fit (Little, 2013).

After defining the final and adequate version of the scale according to the first sub-sample data, the solution’s stability was tested by performing a CFA with the second sub-sample. Finally, as suggested by current guidelines (Dunn et al., 2014), we estimated the composite reliability to verify the reliability of each obtained factor.

#### Generalizability Evidence

Multi-group analyses were performed to collect evidence about the generalizability of the interpretation of the test scores across different subgroups. Specifically, four types of measurement invariance (configural, weak, strong, strict; Dimitrov, 2010) and two types of structural invariance (factor variance and factor mean; Dimitrov, 2010) were tested across subgroups based on relevant variables for the construct under investigation: respondent (mother vs. father), child’s primary caregiver (mother vs. father vs. grandparent), living region (north China vs. south China), as well as family income group (less than 5,000 CNY vs. 5,000–10,000 CNY vs. 10,000–20,000 CNY vs. more than 20,000 CNY).

Measurement invariance is important as it verifies that factorial structure (*configural*), factor loadings (*weak*), intercepts (*strong*), and residuals (*strict invariance*) are equivalent across groups; this equivalence allows for a comparison of PPE scores across groups, being sure that any difference found is a true difference and not a measurement artifact. Furthermore, structural invariance is important as it compares latent factors’ variance (factorial variance) and means (factorial mean invariance), as is usually done through a *t*-test or ANOVA. Structural invariance has an advantage over these two analyses (*t*-test and ANOVA) because the variance and mean comparison are made with the latent score rather than the observed score, which produces an estimation not affected by measurement error (Jiang et al., 2017).

The plausibility of the invariance constraints is tested by comparing a constraint model with a previous one (e.g., comparing the strong invariance model with the weak invariance model) and evaluating how much they differ according to the ΔCFI and the Δχ^2^. Specifically, when testing the measurement invariance, the change in CFI was adopted as it is the recommended method (Cheung and Rensvold, 2002). A negative Δ*CFI* value lower than −0.01 indicates that the two compared models are significantly different (Cheung and Rensvold, 2002). When we tested the structural invariance, we adopted the Δχ^2^ (as suggested by Sorgente and Lanz, 2019) because the change in CFI is not sensitive enough for meaningful change on latent parameters (factor variance, factor mean). Similarly, the χ^2^ statistic is overly sensitive for large numbers of constraints, especially when estimated on large sample sizes (e.g., Marsh et al., 1988); thus, we did not use the cut-off *p* < 0.05 to consider the two compared models significantly different. Specifically, we considered an adjusted *p*-value of 0.001 to reduce the possibility of a type I error (Little, 1997). Finally, to increase the power of the invariance analyses, the analyses were performed on the total sample (*N* = 833).

#### Evidence Based on Relations to Other Variables

As suggested by Zumbo and Chan (2014), this kind of evidence tests the degree of association between the measure being examined and measures representing similar constructs to verify that these associations are consistent with theoretical expectations. According to the current guidelines (e.g., Zumbo, 2005), these associations should be tested within a Structural Equation Model to obtain estimations free from the measurement model. Consequently, to test the association that the PPE scale has with parents’ pro-environmental behaviors and feelings about nature, we ran a Structural Equation Model where we estimated the factorial structures of the three scales and correlated their latent factors.

## Results

### Data Cleaning and Descriptive Analysis

After verifying that the missing data was completely random [Little’s MCAR test: χ^2^(59) = 61.844; *p* = 0.375], we removed 81 monovariate and 55 multivariate outliers. For the remaining 833 participants, we estimated the descriptive statistics for categorical (Table 1) and quantitative (Table 2) variables.

**TABLE 1:** Frequency of categorical items (*N* = 833).

Question	Option 1 (*n*, %)	Option 2 (*n*, %)	Option 3 (*n*, %)	Option 4 (*n*, %)
Who is the respondent?	Mother (636, 76.4%)	Father (171, 20.5%)	Other (26, 3.1%)	
Who is the child’s primary caregiver?	Mother (608, 73.0%)	Father (107, 12.8%)	Grandparent (103, 12.4%)	Other (15, 1.8%)
How much is the family income?	Less than 5,000 CNY (277, 33.3%)	5,000–10,000 CNY (254, 30.5%)	10,000–20,000 CNY (166, 19.9%)	More than 20,000 CNY (136, 16.3%)
In which region does the family live?	North China (620, 77.4%)	South China (213, 25.6%)		

**TABLE 2:** Descriptive statistics of quantitative measures (*N* = 833).

Question	Min	Max	Mean	Standard deviation
Age	25	66	37.07	6.15
Parental participation	1.00	5.00	3.58	0.75
Pro-environment behavior	2.57	5.00	3.95	0.48
Connectedness with nature	1.83	5.00	4.18	0.61

### Score Structure Evidence

After creating two random sub-samples (417 vs. 416 cases), we performed an item analysis and EFA with the first sub-sample. All the PPE scale items presented a normal distribution and a response rate of 100% (see Table 3).

**TABLE 3:** Missing data and distribution for the 18 items of the parental participation in the environment scale (*N* = 417).

	Missing	Min	Max	Mean	*SD*	Skewness	Kurtosis
Item 1	0%	1	5	3.57	0.96	–0.09	–0.57
Item 2	0%	1	5	3.65	0.92	–0.04	–0.78
Item 3	0%	1	5	2.51	1.01	0.49	0.06
Item 4	0%	1	5	2.12	1.02	0.79	0.36
Item 5	0%	1	5	3.22	0.97	0.14	–0.19
Item 6	0%	1	5	3.47	1.05	–0.23	–0.62
Item 7	0%	1	5	3.06	1.10	0.18	–0.54
Item 8	0%	1	5	3.25	1.03	–0.06	–0.50
Item 9	0%	1	5	3.51	0.97	–0.14	–0.36
Item 10	0%	1	5	3.59	0.85	0.05	–0.42
Item 11	0%	1	5	3.39	0.90	0.22	–0.43
Item 12	0%	1	5	2.75	1.08	0.20	–0.42
Item 13	0%	1	5	3.75	0.80	–0.11	–0.25
Item 14	0%	1	5	3.80	0.87	–0.26	–0.44
Item 15	0%	1	5	3.46	1.03	–0.17	–0.52
Item 16	0%	1	5	3.69	0.89	–0.22	–0.31
Item 17	0%	1	5	3.41	1.00	–0.17	–0.42
Item 18	0%	1	5	3.51	0.95	–0.08	–0.50

Consequently, all the scale items were used to run the EFA. Initial or extraction communality was always higher than 0.40, so items shared sufficient variance. The EFA solution showed two factors explaining 50.92 and 4.36% of items’ variance. Looking at the factor loadings (see Table 4), we decided to remove items 5, 6, 7, 8, and 12, as they had similar factor loadings on both factors (i.e., they could not discriminate between different dimensions). Item 3 (“Take your children to the public area to pick up rubbish, mineral water bottles, etc.”) and 4 (“Take your children to clean up pet feces in the community”) were the only two items loading only on the second factor. We decided to delete this second factor (and consequently items 3 and 4) for the following reasons: (1) factors composed of only two items should be avoided to have a psychometrically strong scale (Marsh et al., 1998); (2) the content of the two items composing this factor does not represent any specific aspect of the “parental participation in the environment” construct that is theoretically meaningful/relevant; and (3) this factor was able to explain a very low portion of items’ variance (i.e., 4.36%).

**TABLE 4:** Results of the exploratory factor analysis with the first sub-sample (*N* = 417).

	Factor 1	Factor 2
Item 1	**0.532**	0.233
Item 2	**0.561**	0.264
Item 3	−0.016	**0.746**
Item 4	0.019	**0.750**
Item 5	0.484	0.398
Item 6	0.391	0.378
Item 7	0.408	0.456
Item 8	0.416	0.382
Item 9	**0.710**	0.050
Item 10	**0.918**	–0.162
Item 11	**0.866**	–0.054
Item 12	0.297	0.311
Item 13	**0.840**	–0.132
Item 14	**0.756**	–0.018
Item 15	**0.533**	0.174
Item 16	**0.638**	0.086
Item 17	**0.641**	0.143
Item 18	**0.811**	–0.035

*Bold values: the items are loaded into a single factor; and the factor loadings are above 0.30.*

Thus, we ran a new EFA excluding items 3, 4, 5, 6, 7, 8, and 12. The new factorial solution was adequate because it produced one factor that explained 55.72% of the variance of the 11 items included in the analysis and because all these items had factor loadings higher than 0.60 (range from 0.67 to 0.82). We then estimated Cronbach alpha of this factor (α = 0.93), and we found that each of the 11 items, if excluded, did not increase this alpha value. In other words, all the items gave a positive contribution to the score’s reliability, so we decided to retain them all.

Finally, using the first sub-sample, we ran a Confirmatory Factor Analysis to check for correlations among items’ residuals. Fit indices of the model [χ^2^(44) = 180.51, *p* < 0.001; RMSEA = 0.086 (0.073, 0.100); CFI = 0.936; SRMR = 0.038] confirmed the mono-dimensional structure of the scale but also showed that the model was improvable. In particular, when checking the model’s modification indices, we found that adding a correlation between item 1 and item 2’s residuals would have reduced the χ^2^-value of 50.59; adding a correlation between item 10 and item 11’s residuals would have reduced the χ^2^-value of 47.57. Other possible modifications were much less relevant (χ^2^-value reduction < 12). We speculated that the correlation between item 1 (“Separate the trash with your children”) and item 2 (“Repair toys or other daily necessities together with your children, which not only saves household expenses for purchasing new products but also reduces household waste.”) was due to both items referring to the trash/waste produced in their own house. In the same way, the correlation between item 10 (“When going out to play in the wild, teach children to recognize various plants and understand their relationship with human life.”) and item 11 (“Take children to learn about various insects in various seasons outdoors and understand their living habits.”) was due to both items referring to teaching children about elements of nature. To avoid redundant items (and the consequent need for correlations among items’ residuals), we removed one item from each couple of redundant items. In particular, we removed items 1 and 10 from the scale, as they were the items with the lowest factor loadings and communalities between the two similar items. After removing these two items, we tested the CFA of the revised PPE scale that contained only nine items (items 2, 9, 11, 13, 14, 15, 16, 17, and 18), and we obtained optimal fit indices [χ^2^(27) = 63.44, *p* < 0.001; RMSEA = 0.057 (0.039, 0.075); CFI = 0.977; SRMR = 0.027] as well as modification indices (χ^2^value reduction for any possible modification < 10), which suggested to retain this version of the scale as definitive.

After defining the final version of the PPE scale with the first sub-sample, we tested the same model again with the second sub-sample (*N* = 416) to verify if the solution we found would be stable when tested on other participants as well. The fit indices of the CFA from the second sub-sample [χ^2^(27) = 89.56, *p* < 0.001; RMSEA = 0.075 (0.058, 0.092); CFI = 0.966; SRMR = 0.029] as well as the high factor loadings (ranging from 0.74 to 0.84) confirmed that the model was stable, so we proceeded estimating the composite reliability for this scale score (ω = 0.93). In sum, the final version of the PPE scale is composed of nine items (see Supplementary Appendix A) that load on a single and highly reliable factor.

### Generalizability Evidence

Using the final version of the nine-item PPE scale and the entire sample (*N* = 833), we tested both measurement invariance and structural invariance across groups based on the following variables: respondent, primary caregiver, family income, and living region. After verifying that the expected factorial structure also had a good fit on the total sample (see Table 5), we proceeded with the first comparison (respondent). Results (see Table 5) indicate that the scale has the same factorial structure, factor loadings, intercepts, residuals, factor variance, and factor mean when either the mother or the father fills out the questionnaire.

**TABLE 5:** Measurement and structural invariance of the parental participation in the environment scale.

	χ^2^	*df*	*p*	RMSEA (90% CI)	CFI	SRMR	Δχ^2^	Δ*df*	*p*	Δ*CFI*
Total sample	128.10	27	<0.001	0.067 (0.056, 0.079)	0.970	0.026				
**Respondent** (636 mothers; 171 fathers)										
Configural invariance	151.19	54	<0.001	0.067 (0.054, 0.080)	0.969	0.030				
Weak invariance	167.72	62	<0.001	0.065 (0.053, 0.077)	0.967	0.047				–0.002
Strong invariance	184.78	70	<0.001	0.064 (0.053, 0.075)	0.964	0.052				–0.003
Strict invariance	205.49	79	<0.001	0.063 (0.053, 0.074)	0.960	0.062				–0.004
Factor variance	206.43	80	<0.001	0.063 (0.052, 0.073)	0.960	0.067	0.34	1	0.559	
Factor mean	208.30	81	<0.001	0.062 (0.052, 0.073)	0.960	0.075	1.66	1	0.197	
**Primary caregiver** (608 mothers; 107 fathers; 103 grandparents)										
Configural invariance	194.01	81	<0.001	0.072 (0.059, 0.084)	0.968	0.032				
Weak invariance	225.64	97	<0.001	0.070 (0.058, 0.082)	0.964	0.064				–0.004
Strong invariance	245.10	113	<0.001	0.065 (0.054, 0.077)	0.963	0.065				–0.001
Strict invariance	268.00	131	<0.001	0.062 (0.051, 0.072)	0.962	0.064				0
Factor variance	273.14	133	<0.001	0.062 (0.052, 0.073)	0.961	0.086	5.36	2	0.068	
Factor mean	274.91	135	<0.001	0.062 (0.051, 0.072)	0.961	0.088	1.07	2	0.586	
**Family income** (277 less than 5,000 CNY; 254 between 5,000 and 10,000 CNY; 166 between 10,000 and 20,000 CNY; 136 more than 20,000 CNY)										
Configural invariance	226.03	108	<0.001	0.072 (0.059, 0.086)	0.967	0.034				
Weak invariance	266.87	132	<0.001	0.069 (0.057, 0.081)	0.963	0.065				–0.004
Strong invariance	357.01	156	<0.001	0.079 (0.068, 0.089)	0.943	0.089				–0.020
- freeing item 13-G1	336.14	155	<0.001	0.075 (0.064, 0.086)	0.949	0.082				–0.014
- freeing item 18-G4	326.20	154	<0.001	0.073 (0.062, 0.084)	0.952	0.078				–0.011
- freeing item 13-G2	316.80	153	<0.001	0.072 (0.061, 0.083)	0.954	0.074				–0.009
Strict invariance	355.25	168	<0.001	0.073 (0.063, 0.084)	0.947	0.088				–0.007
Factor variance	358.11	171	<0.001	0.072 (0.062, 0.083)	0.947	0.094	1.50	3	0.682	
Factor mean	362.22	174	<0.001	0.072 (0.062, 0.083)	0.947	0.100	3.75	3	0.289	
**Living region** (620 in North China; 213 in South China)										
Configural invariance	169.92	54	<0.001	0.072 (0.060, 0.084)	0.966	0.030				
Weak invariance	190.66	62	<0.001	0.071 (0.059, 0.082)	0.963	0.048				–0.003
Strong invariance	210.01	70	<0.001	0.069 (0.059, 0.080)	0.959	0.048				–0.004
Strict invariance	215.80	79	<0.001	0.064 (0.054, 0.075)	0.960	0.055				0.001
Factor variance	216.45	80	<0.001	0.064 (0.054, 0.074)	0.960	0.055	0.27	1	0.601	
Factor mean	249.702	81	<0.001	0.071 (0.061, 0.081)	0.951	0.098	38.03	1	<0.001	

*χ^2^, chi-square; df, degree of freedom; RMSEA, Root Mean Square Error of Approximation; CI, Confidence Interval; CFI, Comparative Fit Index; SRMR, Standardized Root Mean Square Residuals.*

A full equivalence was found for the second comparison too (primary caregiver). Despite who the primary caregiver was (mother, father, grandparent), the PPE scale worked equally well. In addition, the variance and mean level of parental participation in pro-environmental activities within the parent-child relationship were equivalent.

Instead, we did not find a full measurement invariance when comparing the scale across families with different income levels (see Table 5). In particular, we found that the intercepts of item 13 (“Take children to walk and play in natural environments, such as parks and forests, so that children can get closer to nature”) was lower in group 1 (3.613) and group 2 (3.739) than it was in groups 3 and 4 (3.910 for both groups). This means that parents with lower incomes (groups 1 and 2) produced, on average, lower scores to this item than parents with higher incomes (groups 3 and 4), although they had the same level of parental involvement. Furthermore, we found that the intercept of item 18 (“Take children to museums to learn about various endangered or extinct animals and let them understand the relationship between the environment and animal survival.”) was higher for parents from the highest income group (group 4) than it was for the other three groups (3.704 vs. 3.467). These non-equivalent intercepts could depend on the fact that taking children to parks and forests (item 13) as well as to museums (item 18) can be expensive, so parents with lower incomes do it less frequently despite their level of parental participation in pro-environmental activities.

Even though we found two items out of nine that were not fully invariant across income groups, we had a sufficient number of invariant items (80%; Dimitrov, 2010) to proceed with structural invariance. We found that factor variance and mean did not differ for the four family income groups. In other words, the level of parental participation was not affected by the parents’ income levels—it only affected specific pro-environmental activities that imply costs.

Finally, we tested the scale invariance across families living in North vs. South China. Results suggest that the scale worked equivalently for the two groups (measurement invariance), but parents living in the South reported a lower level (factor mean = −0.521) of parental participation than parents living in the North.

### Evidence Based on Relations to Other Variables

We ran a Structural Equation Model in which one factor for each construct (parents’ PPE, pro-environmental behavior, connectedness with nature) was estimated and the correlations between these factors were required. The model had good fit indices [χ^2^(365) = 1194.25, *p* < 0.001; RMSEA = 0.052 (0.049, 0.056); CFI = 0.921; SRMR = 0.048] and indicated that PPE was significantly (*p* < 0.001) associated with both parents’ pro-environmental behavior [*r* = 0.743 (95% Confidence Interval = 0.700–0.786)] and connectedness with nature [*r* = 0.484 (95% Confidence Interval = 0.417–0.552)], as expected according to the literature. Both relationships were positive in direction and medium/strong in effect size, as expected according to the literature (e.g., Nisbet et al., 2009; Katz-Gerro et al., 2020).

## Discussion

Parents play a crucial role in children’s development. Recent studies have revealed that parents’ environmental behaviors and values can convey to children (Grønhøj and Thøgersen, 2009, 2012); however, this intergenerational transmission may depend on parental socialization, such as parental participation (Grusec and Davidov, 2019). Parents who participate in family activities with their children will reinforce their children’s understanding of the values of the activities and enable them to learn from them (Rogoff et al., 2007; Grusec and Davidov, 2019). We believe that parental participation should also influence intergenerational transmission in an environmental domain (e.g., pro-environmental behavior and worldviews). Several studies have explored the connection between parental participation and intergenerational transmission as they relate to pro-environmental behavior (e.g., Katz-Gerro et al., 2020; Jia and Yu, 2021); however, there was no validated instrument available to assess the level of PPE with children in these studies. Thus, in line with the previous studies, we developed the initial items of parent-child environmental activities (Jia and Yu, 2021), as well as validated these items and developed the PPE scale in a large sample of Chinese parents.

The PPE scale was developed *via* a two-step interview. In the pilot study (Jia and Yu, 2021), we asked parents to list environmental activities they had been involved in with their children. Although past studies have used this method successfully to create an initial list of items (e.g., Krettenauer et al., 2016), it was ineffective in the current study because many parents provided repetitive activities (e.g., recycling, picking up garbage, saving energy) and ones without any further elaboration. After consulting with a local Chinese researcher, we added a second step to the interview—small group discussions. The local researcher also suggested grouping parents whose children were in the same summer schools together so that they would not have to share information with a stranger (e.g., a research assistant). In addition to synchronous discussion, these parents also had an option to engage in the discussion asynchronously (e.g., leave a message and reply to messages in their free time). Research assistants moderated the group chats with minimal interference. Many new activities and rationales emerged from the small group discussions.

To ensure our scale was appropriate for use in China, we validated the PPE scale among a representative group of Chinese parents. Following the scale validation procedure (Sorgente and Lanz, 2019), we randomly divided the large sample (*N* = 833) into two sub-samples. We started with exploratory factor analysis (EFA) and found one common factor with 11 items. Then we conducted a confirmatory factor analysis (CFA) and removed two redundant items (e.g., ones with the lowest factor loadings and communalities between the two similar items). The same CFA model with nine items was repeated for the second half of the sample. The results confirmed the stability of the model and suggested retaining this version of the scale as definitive.

Generalizability evidence of the nine-item PPE scale was obtained using the entire sample. The scale’s measurement invariance was found for respondents (mothers and fathers who responded to the survey), primary caregivers (mothers, fathers, and grandparents who were the primary caregivers), and regions (North and South). This generalizability is an advantage of the newly developed scale because environmental activities can apply equally well regardless of who fills out the survey, who the primary caregiver is, and where the family locates. It suggests that any mean-level similarities or differences should not be due to measurement errors (Sorgente and Lanz, 2019).

Although the current study revealed that fathers and mothers had been equally involved with the child in the PPE scale, it would be interesting to examine the effectiveness of the parental contributions to children’s outcomes toward the environment (e.g., environmental values and behaviors). Past research has suggested paternal involvement positively affects children’s psychological outcomes (Flouri and Buchanan, 2003; Videon, 2005); maternal and grandparent involvements have also been positively associated with children’s prosocial behavior (Profe and Wild, 2017). A qualitative study found that environmental activists recalled early environmental stories about family traditions, such as grandparent involvement in daily environmental activities (Jia et al., 2015). However, no study has investigated the grandparent-grandchild relationships regarding environmental behavior. Unfortunately, the current study cannot test these relationships. Future studies should examine the role of grandparents, especially if they live with their children and participate in their daily activities. The PPE scale may provide a first step to disentangle these complex dyadic relationships.

In addition, we found that parents living in North China reported a greater PPE score than parents from South China. One recent study might support this regional difference, as it found that residents in the North (e.g., Heilongjiang) scored higher in environmental values than residents in the South (e.g., Ningxia; Yu, 2014). Possible reasons for the regional differences may be due to complex cultural and economic differences. Cities in North China typically are dominated by industrial development, where residents may have a high awareness of environmental pollution (Jia and Yu, 2021). In contrast, cities in South China are rooted in agricultural traditions, where land and resources are abundant. Consequently, residents in South China may downplay environmental destructions (Jiang and Zhang, 2005; Yu, 2014). However, we cannot overgeneralize this finding because neither the current study nor the past study selected representative samples across northern and southern Chinese cities or examined these economic factors. Future studies should consider the region, cultural practice, and economic development as factors of individual pro-environmental behaviors, values, and involvement in children’s environmental activities.

Not surprisingly, we did not find a full measurement invariance when examining the PPE scale across families’ income levels. In particular, parents with relatively low incomes reported a lower score on the two PPE items related to cost (e.g., take children to museums or national parks) than the parents with relatively high incomes. A large body of research has indicated SES plays an important role in the accessibility of green spaces (Cohen et al., 2016; Tillmann et al., 2018). This relationship also extends to parents’ investment and engagement in green/environmental activities with children. Although the PPE scale was not fully invariant across parental incomes, the nine-item PPE scale still measured the construct in a way that is equivalent enough to allow PPE score comparisons across income groups (Dimitrov, 2010).

Finally, we found the PPE scale positively related to parents’ pro-environmental behavior and connectedness with nature. The general literature supports this relational evidence (e.g., Nisbet et al., 2009; Krettenauer et al., 2020) and the positive association with parental involvement. Future research should examine the directionality of the association: do parents’ and children’s pro-environmental behaviors and connectedness with nature predict parent-child mutual involvement (Gentina and Muratore, 2012)?

The present study has several limitations. First, although parents from multiple Chinese cities were interviewed in the preliminary study (Jia and Yu, 2021), the sample was not representative. Future research should systematically investigate regional differences and randomly select samples from northern and southern cities in China to develop the initial items.

Second, parents were recruited from their children’s summer schools. These parents may highly value education and have extra resources to send their children to the costly private-for-profit summer schools in various subjects (e.g., English, sciences, music); thus, these parents may be more likely to be involved with their children academically and socially than parents who lack these resources. As a result, it is necessary to validate the PPE scale in disadvantaged families.

Third, we presumed that PPE would predict children’s environmentalism (e.g., pro-environmental behavior, values, and beliefs). However, the present study only established the relationship between PPE and parents’ environmental behavior—not with children’s behavior. It seems critical to collect parent-child dyadic data and demonstrate the external validity that parents’ PPE predicts children’s environmentalism concurrently and longitudinally.

In addition, although the current study indicated that data were collected from parents whose children attended summer elementary schools (1st to 6th grades), it did not specify the children’s exact age. Research has shown that children’s pro-environmental behaviors decline from childhood to adolescence. Further research should focus on age differences and determine possible causal influences for the decline.

Lastly, the current PPE scale was based on only one domain of parental socialization—parental participation. The scale did not consider other aspects of parental socialization, such as parent-child relationships, parenting styles, and parental control. Future studies should investigate these domains and develop/validate the measures accordingly.

Despite these limitations, the present study adds to a growing literature on parental socialization (e.g., parental participation) in an environmental context (Grusec, 2011; Katz-Gerro et al., 2020). The PPE scale was developed from parents’ perspectives and validated in a relatively diverse sample in China. Full measurement invariance of the nine PPE items was found across different respondents, caregivers, and regions. An adequate invariance (7 out of 9 items) was obtained across families’ income levels. The PPE scale is also positively related to other environmental variables (pro-environmental behavior and connectedness with nature). Although the PPE scale cannot be generalized to different cultures (or other regions in China), it provides a novel measure to examine the role of parental socialization in children’s environmentalism.

## Data Availability Statement

The raw data supporting the conclusions of this article will be made available by the authors, without undue reservation.

## Ethics Statement

The studies involving human participants were reviewed and approved by the Ethics Review and Research Committee at the College of Education in Harbin Normal University, China. The patients/participants provided their written informed consent to participate in this study.

## Author Contributions

FJ: research conception, experiment design, data analyses, draft initial writing, and revise and draft the final version. AS: data analyses, draft initial writing, and revise and draft the final version. HY: experiment design and data collection. All authors contributed to the article and approved the submitted version.

## Conflict of Interest

The authors declare that the research was conducted in the absence of any commercial or financial relationships that could be construed as a potential conflict of interest.

## Publisher’s Note

All claims expressed in this article are solely those of the authors and do not necessarily represent those of their affiliated organizations, or those of the publisher, the editors and the reviewers. Any product that may be evaluated in this article, or claim that may be made by its manufacturer, is not guaranteed or endorsed by the publisher.
