# Supplementary material for: Parental Participation in the Environment: Scale Validation Across Parental Role, Income, and Region
Source: Front Psychol. 2022 Mar 4;13:788306. doi: 10.3389/fpsyg.2022.788306 (PMC8934388; doi:10.3389/fpsyg.2022.788306)
Supplement: Supplementary file 1 [file Data_Sheet_1.docx]

**Appendix A: Detail Descriptions in Development of the Items**

Forty-five Chinese parents were invited for brief online interviews. All were primary caregivers (Mage = 36.38 years; SD = 3.68 ranging from 29 to 43 years; 79% mother, 21% father). The parents were contacted from local summer schools, which were selected via personal contacts. Consent forms were obtained, and a unique ID was assigned to each parent. The study was approved by the Ethics and Research Board at the third author’s university in China.

The interviews took two days to complete and involved two steps. In the first step, parents participated in an individual interview where they were asked to list environmentally friendly activities they had actively been involved in with their children. They were asked to keep track of these activities so that they could share them with other parents in a group discussion later. In the second step, all parents were divided into small groups that matched their children’s summer schools. In these groups, parents were asked to discuss their list of environmental involvements with the other parents. All parents could participate in the discussion synchronously (e.g., live chat) and asynchronously (e.g., reply by text or audio message). Research assistants moderated the group discussions. Please note that parents in the group discussions typically knew each other because their children were in the same summer schools. By interacting with familiar parents, they began to think of new activities they had not thought about in the individual interview. Parents could also generate more results in the group discussions because they could reply to message, join conversations, and chat with other parents at any time.

A total of 96 activities were collected during the two-step interview. Similar to a previous study’s procedure (Krettenauer et al., 2016; Jia et al., 2019), three steps were used to narrow down the activities. First, we separated conversations into multiple items. Second, we removed the activities that were only mentioned once or twice. Third, we combined similar activities into one item. A narrowed list of 18 activities was generated from the procedure. Chinese research assistants and the third author reworded the activities and created an 18-item PIE scale in Mandarin Chinese (see Appendix A). For example, many parents mentioned “taking children to community park, provincial parks, or forests”, followed up with activities such as “play, walk, running etc” and motivations “connect with nature, get closer to nature.” These activities were reworded as “take children to walk and play in natural environments such as parks and forests, so that children can get closer to nature” (please note that Google Translation translated Mandarin Chinese for a demonstration purpose). The 18 items were sent back to the parents’ group discussions. After offering the parents a chance to make any changes, some minor language and logical issues were identified. However, no new activities were added by the parents. The language and face validity of the 18 items were checked and confirmed by the research assistants (graduate students), authors, and two other colleagues in China.

Forty-five Chinese parents were invited for brief online interviews. All were primary caregivers (Mage = 36.38 years; SD = 3.68 ranging from 29 to 43 years; 79% mother, 21% father). The parents were contacted from local summer schools, which were selected via personal contacts. Consent forms were obtained, and a unique ID was assigned to each parent. The study was approved by the Ethics and Research Board at the third author’s university in China.

The interviews took two days to complete and involved two steps. In the first step, parents participated in an individual interview where they were asked to list environmentally friendly activities they had actively been involved in with their children. They were asked to keep track of these activities so that they could share them with other parents in a group discussion later. In the second step, all parents were divided into small groups that matched their children’s summer schools. In these groups, parents were asked to discuss their list of environmental involvements with the other parents. All parents could participate in the discussion synchronously (e.g., live chat) and asynchronously (e.g., reply by text or audio message). Research assistants moderated the group discussions. Please note that parents in the group discussions typically knew each other because their children were in the same summer schools. By interacting with familiar parents, they began to think of new activities they had not thought about in the individual interview. Parents could also generate more results in the group discussions because they could reply to message, join conversations, and chat with other parents at any time.

A total of 96 activities were collected during the two-step interview. Similar to a previous study’s procedure (Krettenauer et al., 2016; Jia et al., 2019), three steps were used to narrow down the activities. First, we separated conversations into multiple items. Second, we removed the activities that were only mentioned once or twice. Third, we combined similar activities into one item. A narrowed list of 18 activities was generated from the procedure. Chinese research assistants and the third author reworded the activities and created an 18-item PIE scale in Mandarin Chinese (see Appendix A). For example, many parents mentioned “taking children to community park, provincial parks, or forests”, followed up with activities such as “play, walk, running etc” and motivations “connect with nature, get closer to nature.” These activities were reworded as “take children to walk and play in natural environments such as parks and forests, so that children can get closer to nature” (please note that Google Translation translated Mandarin Chinese for a demonstration purpose). The 18 items were sent back to the parents’ group discussions. After offering the parents a chance to make any changes, some minor language and logical issues were identified. However, no new activities were added by the parents. The language and face validity of the 18 items were checked and confirmed by the research assistants (graduate students), authors, and two other colleagues in China.

**Appendix B: Parental Participation in the Environment (PPE) Scale Items**

| Items in Chinese | Items in English |
| --- | --- |
| 1 和孩子一起给垃圾分类。 | 1 Sort the trash with your child. |
| *2 和孩子一起维修玩具或其它生活用品，既节约购买新产品的家庭开支，有减少生活垃圾。 | *2 Repair toys and household supplies with your child to reduce household expenses and waste. |
| 3 带孩子到公共区域捡垃圾、矿泉水瓶。 | 3 Pick up trash and plastic water bottles in public areas with your child. |
| 4 带孩子清理社区中的宠物粪便。 | 4 Clean up pet feces in your community with your child. |
| 5 和孩子一起做环保手工，变废为宝，制作实用的家庭生活用品。 | 5 Make environmentally friendly crafts with your child by turning waste into treasure with household supplies. |
| 6 鼓励孩子在家庭中积攒可卖钱的废品，比如废纸, 水瓶等，然后用卖来的钱做更有意义的事情。 | 6 Collect sellable waste products with your child, such as paper, water bottles, etc., and then use the income for more meaningful activities. |
| 7 和孩子一起动手动脑，开发生活中的垃圾为肥料, 比如淘米水浇花，果皮，鱼刺发酵后可做肥料。 | 7 Do hands-on activities with your child and teach them how to use garbage that can be fermented as fertilizer (e.g., rice water, peels, and fish bones). |
| 8 让孩子担任一段时间的节能小卫士，每天负责检查家里的不关灯，水龙头没关等浪费行为。 | 8 Teach your children to conserve energy by turning off the lights and AC at home. |
| *9 协助孩子养育一种动物或植物，在伴随它们成长的过程中，培养对自然的热爱。 | *9 Raise an animal or plant with your child as they grow up and instill a desire to be part of nature. |
| 10 去野外游玩的时候教孩子认识各种植物，并了解它们与人类生活的关系。 | 10 Take your child on an outdoor adventure and teach them how to identify plants as well as understand how they relate to humans. |
| *11 带孩子认识户外各个季节的各种昆虫，了解他们的生活习性。 | *11 Learn about various insects in various seasons outdoors with your child and understand their living habits. |
| 12 带孩子到户外与动物互动，如给野生动物或者流浪的动物喂食。 | 12 Provide your child with the opportunity to interact with animals. |
| *13 带孩子去公园、森林等自然环境中散步, 游戏, 让孩子更亲近自然。 | *13 Walk and play in natural environments with your child, such as parks and forests, to help them get closer to nature. |
| *14 教孩子循环使用购物袋，去购物的时候提醒孩子养成自备购物袋的习惯，较少所料垃圾的产生。 | *14 Recycle shopping bags with your child and encourage them to bring their own shopping bags when going shopping to reduce waste. |
| *15 外出就餐，让孩子自备餐具，减少使用一次性筷子及其它餐具。 | *15 Encourage your child to bring their own reusable containers when eating out and reduce the use of disposable chopsticks. |
| *16 出行的时候，尽量带孩子选择绿色出行方式，并解释原因。 | *16 Discuss with your children the importance of choosing eco-friendly modes of travel. |
| *17 购买食品的时候向孩子讲解有机食品等相关知识。 | *17 Explain organic food and other related knowledge to children when buying food. |
| *18 带孩子去博物馆认识各种濒危甚至已经灭绝的动物，让他们了解环境与动物生存的关系。 | *18 Take children to Zoos and museums to learn about various endangered or even extinct animals and let them understand the relationship between the environment and animal survival. |

Note. These items were translated and back-translated by two bilingual (Chinese-English) research assistants.

* Indicates the final nine items in the PPE scale.

Some unvalidated items in the scale have been published in a previous article (Jia & Yu, 2021). We have obtained the permission from the copyright owner to use, modify, and list all the items in the current study.

Jia, F., & Yu, H. (2021). Brief data report on parent-child pro-environmental engagement across five cities in China. *Data in Brief*. *36*, 106970. <https://doi.org/10.1016/j.dib.2021.106970>

**Appendix C: Connectedness with Nature (Liu & Chen, 2018)**

The Connectedness with Nature scale was adapted from Collado, Staats and Corraliza (2013) and used in a Chinese study by Liu and Chen (2018). The Chinese versions of the measure are available in Liu & Chen’s (2018) article.

Collado, S., Staats, H., & Corraliza, J. A. (2013). Experiencing nature in children's summer camps: Affective, cognitive and behavioural consequences. *Journal of Environmental Psychology*, *33*, 37-44.

Liu, W., & Chen, J. (2018). Influence of pupils’ cognitive and emotional environmental attitudes on their environmental behavior – Taking Kunming city as an example. *Journal of Green Science and Technology,* *13*, 281-285. DOI:10.16663/j.cnki.lskj.2018.13.120

**Appendix D: Pro-Environmental Behavior (Krettenauer, 2017))**

The Pro-Environmental Behavior scale was developed by Krettenauer (2017) to assess the levels of parents’ pro-environmental behaviors and was used in a Chinese study (Krettenauer et al., 2021). The items of the measure are available in Krettenauer’s (2017) article.

Krettenauer, T. (2017). Pro‐environmental behavior and adolescent moral development. *Journal of Research on Adolescence*, 27(3), 581-593. https://doi.org/10.1111/jora.12300

Krettenauer, T., Wang, W., Jia, F., & Yao, Y. (2020). Connectedness with nature and the decline of pro-environmental behavior in adolescence: a comparison of Canada and China. *Journal of Environmental Psychology*, 71, 101348. https://doi.org/10.1016/j.jenvp.2019.101348
